# 

**Published:** 1859-10

**Authors:** 


### OHIO COLLEGE OF DENTAL SURGERY.

A preliminary course of lectures and demonstrations will commence in this institution on the 18th of October next. There will be one or two lectures each day, and three or four clinics each week from that till the first Monday of Movember, when the regular course will commence.

Much addition is being made to the several departments, especially the mechanical.

The laboratory is receiving a new supply of fixtures. Instruction will be given in all kinds and styles of mechanical work—all at least that have any intrinsic value. The Faculty are desirous that the students all be in attendance by the 18th of October, so far as possible, for it is the intention to begin in earnest, and work so throughout the session. T.

### NEW YORK DENTAL JOURNAL,

IS ISSUED QUARTERLY,

### PRICE, $2.00 PER ANNUN, IN ADVANCE.

Each number contains sixty-four pages of matter, original and selected. A few pages of Advertisements will be received, terms as follows:

For one page, one year........$25 00

" ½ " " ........ 15 00

" ¼ " " ........ 10 00

And for shorter periods in proportion.

All communications, subscriptions, and business notes of any kind, must be addressed to the

**"NEW YORK DENTAL JOURNAL,"**

No. 640 Broadway, New York.

